# Comprehensive Identification of SUMO2/3 Targets and Their Dynamics during Mitosis

**DOI:** 10.1371/journal.pone.0100692

**Published:** 2014-06-27

**Authors:** Julie Schou, Christian D. Kelstrup, Daniel G. Hayward, Jesper V. Olsen, Jakob Nilsson

**Affiliations:** The Novo Nordisk Foundation Center for Protein Research, Faculty of Health and Medical Sciences, University of Copenhagen, Copenhagen, Denmark; Virginia Tech, United States of America

## Abstract

During mitosis large alterations in cellular structures occur rapidly, which to a large extent is regulated by post-translational modification of proteins. Modification of proteins with the small ubiquitin-related protein SUMO2/3 regulates mitotic progression, but few mitotic targets have been identified so far. To deepen our understanding of SUMO2/3 during this window of the cell cycle, we undertook a comprehensive proteomic characterization of SUMO2/3 modified proteins in mitosis and upon mitotic exit. We developed an efficient tandem affinity purification strategy of SUMO2/3 modified proteins from mitotic cells. Combining this purification strategy with cell synchronization procedures and quantitative mass spectrometry allowed for the mapping of numerous novel targets and their dynamics as cells progressed out of mitosis. This identified RhoGDIα as a major SUMO2/3 modified protein, specifically during mitosis, mediated by the SUMO ligases PIAS2 and PIAS3. Our data provide a rich resource for further exploring the role of SUMO2/3 modifications in mitosis and cell cycle regulation.

## Introduction

Proper progression through mitosis depends on tight regulation of protein activities in a spatial and temporal manner. This regulation is mainly achieved at the level of post-translational modifications (PTMs), as this is a rapid way of changing protein activities. Although it is clear that phosphorylation of proteins by mitotic kinases plays an important role, other PTMs have been implicated in mitotic regulation, but remain poorly explored [1].

The modification of proteins with the small ubiquitin-related modification SUMO does not target proteins for degradation, but instead acts to regulate the activity of proteins. Four different variants of SUMO exist in the human genome but only SUMO 1–3 appear to be expressed [2]. SUMO2 and SUMO3 are almost identical and they are therefore referred to as SUMO2/3 [3]. Similar to ubiquitin, the very C-terminal glycine residue of SUMO is conjugated to lysine residues of target proteins. This is catalyzed by a SUMO ligase in conjunction with Ubc9, which is the only E2 enzyme of the SUMO pathway [4]. A number of SUMO ligases have been described including the PIAS 1–4 proteins [5], [6] and their activity is counterbalanced by a set of deSUMOylating proteins referred to as the SENPs [7].

The importance of the SUMO pathway for mitotic progression is highlighted by the fact that the genetic removal of Ubc9 results in errors during chromosome segregation, and dominant negative Ubc9 prevents the metaphase to anaphase transition in frog extracts [8], [9]. Mechanistic insight into the regulation of mitosis by SUMOylation has been achieved through the identification of SUMOylated proteins. Examples being the modification of Topoisomerase II by SUMO2/3, to localize it to centromeres [10]–[12] or the modification of Nuf2 and BubR1 with SUMO2/3 to act as a scaffold for recruiting CENP-E to kinetochores [13]. Despite the importance of the SUMO pathway in mitotic regulation, a comprehensive characterization of targets at close to physiological conditions has not been performed.

Affinity purification of SUMOylated proteins coupled with mass spectrometry is an efficient way of identifying novel targets [14]–[16], but given that SUMOylated proteins are scarce, their identification is still difficult. This is even more of an issue under normal physiological conditions, such as mitosis, where the levels of SUMOylated proteins are very low [13]. Here, we describe an efficient purification strategy of SUMO2/3 modified proteins from mitotic cells, using a tandem affinity purification strategy of FLAG-His tagged SUMO2 expressed at endogenous levels. This strategy allowed the reproducible identification of more than 200 targets. In addition, by combining it with cell synchronization procedures and quantitative mass spectrometry, the dynamics of SUMO2/3 modifications as cells progressed out of mitosis were determined. This revealed that numerous transcription factors were modified by SUMO2/3 as cells progressed out of mitosis while RhoGDIα was one of a few proteins that were highly SUMO2/3 modified during mitosis in a PIAS2 and PIAS3 dependent manner.

The methods and results described here provide useful tools and resources to further explore the SUMO pathway in mitosis and more widely during the cell cycle.

## Materials and Methods

### Cloning and generation of stable cell lines

His-SUMO2 and His-SUMO2ΔGG were amplified from pcDNA3.1 SUMO2 and cloned into BamHI and NotI sites of pcDNA5/FRT/TO 3*FLAG. The C-terminal Q87R mutation was generated by whole plasmid PCR. RhoGDIα was amplified from Invitrogen Ultimate Clone IOH5797 (pENTR221 ARHGDIA) and cloned into BamHI and NotI sites of pcDNA5/FRT/TO 3*FLAG and pcDNA5/FRT/TO 3*FLAG-Venus. RhoGDIα K138R/K141R was generated by whole plasmid PCR. RNAi resistance of generated RhoGDIα constructs towards siRhoGDIα #1 was achieved by mutating ATC CAG CAT ACG TAC AGG (bases 385–402) into ATT CAA CAC ACC TAC CGC by whole plasmid PCR. All constructs were verified by sequencing.

Stable HeLa FRT TRex cell lines, expressing constructs under the control of a doxycycline-inducible promoter, were generated as previously described [17].

### Cell culture and SILAC labeling

HeLa cells were maintained in DMEM supplemented with 10% fetal bovine serum (FBS, HyClone) and 1% Penicillin-Streptomycin (Life Technologies). Parental HeLa FRT TRex cells were cultured under selection with 5 µg/ml Blasticidin HCl (Life Technologies) and 50 µg/ml Zeocin (Life Technologies). Generated stable cell lines were cultured under selection with 5 µg/ml Blasticidin HCl and 200 µg/ml Hygromycin B (Life Technologies). Expression of SUMO2 fusion construct was induced with 1 ng/ml and RhoGDIα with 0.5 ng/ml doxycycline for 48 hours unless otherwise stated. The following drug concentrations were used: Thymidine (Sigma) 2.5 mM, Taxol (Sigma) 100 nM, ZM447439 (Tocris Bioscience) 2 µM.

For quantitative proteomic experiments, cells were grown in SILAC DMEM (PAA) supplemented with 10% dialyzed FBS (PAA), Penicillin-Streptomycin and Glutamax (Life Technologies) and isotope labeled L-arginine and L-lysine. SUMO2ΔGG cells were grown in light “L” SILAC medium supplemented with normal arginine (R0) and lysine (K0) (Sigma). For the medium “M” condition SUMO2 cells were grown in SILAC DMEM supplemented with U-^13^C_6_ arginine (K6) and 4,4,5,5-D_4_ lysine (K4) and for the heavy “H” labeling SUMO2 cells were grown with U-^13^C_6_, U-^15^N_4_ arginine (R10) and U-^13^C_6_, U-^15^N_2_ lysine (K8) (Cambridge Isotope Laboratories). Cells were grown in SILAC medium for at least five divisions before harvest.

### Antibodies

The following antibodies were used at the indicated dilutions for western blot analysis. Mouse anti-FLAG (1∶1000, F3165, Sigma), mouse SUMO2/3 (1∶1000, ab81371, Abcam), rat anti-tubulin (1∶5000, ab6160, Abcam), mouse anti-Vinculin (1∶9000, V9131, Sigma), mouse anti-CyclinB1 (1∶2000, 554177, BD Biosciences), rabbit anti-Aurora A (1∶4000, ab1287, Abcam), rabbit anti-RhoGDIα (1∶2000, sc-360, Santa Cruz Biotechnology) and mouse anti-RhoA (1∶500, sc-418, Santa Cruz Biotechnology).

### Purification of SUMOylated proteins

Mitotic cells were harvested, washed with ice-cold PBS and collected by centrifugation at 500 g at 4°C. Based on the pellet weight, cells were lysed in Gu-HCI lysis buffer (6 M Gu-HCl, 100 mM Na_2_HPO_4_, 10 mM Tris-HCl (pH 8.0), 20 mM imidazole, 10 mM β-mercaptoethanol) – 2 ml lysis buffer per 0.1 g cells. The lysate was filtered through a 0.45 µm sterile filter prior to purification, but in general the lysis procedure and purification using Ni-NTA Superflow beads (Qiagen) was performed as described previously [18]. Multiple rounds of depletion were carried out by incubating flow through from one round with a new portion of Ni-NTA beads. Elution fractions in 6.4 M urea were pooled and, the concentration of urea was reduced by buffer exchange into TBS-T buffer (50 mM Tris, pH 7.4, 300 mM NaCl, 1 mM EDTA, 5% glycerol, 1% TritonX-100) supplemented with 5 mM chloroacetamide, 20 mM N-ethylmaleimide (NEM, Sigma) and EDTA-free protease inhibitor cocktail (Roche) using centrifugal filters (AmiconUltra, Millipore). The buffer-changed sample was incubated with 100 µl anti-FLAG M2 affinity resin (Sigma) for 2 hours at 4°C. The anti-FLAG beads were washed twice in TBS buffer (50 mM Tris, pH 7.4, 150 mM NaCl) and for elution of proteins, LDS sample buffer (Life Technologies) was added to the beads. Before the eluted purified SUMO2-conjugates were collected, beads and sample buffer suspension were boiled for 3 min at 96°C and then centrifuged for 30 sec at 5000 g.

### Large-scale growth and cell synchronization

4*10^7^ cells were seeded with 200 ml medium in one roller bottle (Nunc TufRoll 850 cm^2^), and incubated at 0.1 rpm in a HeraCell incubator with roller bottle device and instrumentation (Thermo Scientific). 24 hours after seeding the rpm were increased to 0.2 and after a further 48 hours the medium was replaced and incubated for an additional 24 hours. Thymidine was then added to a final concentration of 2.5 mM for 20 hours to pre-synchronize cells in S phase. Cells were released from the block by washing twice with 25 ml medium, before adding fresh media. 3 hours after the release, taxol was added to a final concentration of 100 nM. 11 hours after taxol addition, cells arrested in mitosis were harvested by mitotic shake-off. For mitotic checkpoint override, harvested cells and medium were transferred to a new roller bottle, 2 µM ZM447439 was added and the cells were incubated for 1 hour.

### Mass spectrometry analysis

The eluate from the beads was reduced, alkylated and loaded onto an SDS–PAGE gel. The lane was cut into 7 pieces and subjected to in-gel tryptic digestion and subsequent sample desalting and concentration, as described previously [19]. The resulting peptide mixture was analyzed by nano-HPLC–MS/MS using an easy-nLC nanoflow system (Thermo Scientific, Odense, Denmark) connected to a Q Exactive mass spectrometer (Thermo Scientific, Bremen, Germany) through a nano-electrospray ion source. The column length was 150 mm with an inner diameter of 75 µm, and packed in-house with 1.9 µm C_18_ beads (Reprosil-AQ Pur, Dr. Maisch). Each sample was analyzed using a 140 min gradient from 4 to 36% acetonitrile in 0.5% acetic acid. The mass spectrometer was run as described for sensitive acquisition [20]. Data analysis was performed in the MaxQuant environment version 1.3.9.9 using default settings configured for triple SILAC and the Andromeda search engine [21]. The database searched was the UniProt complete human proteome, release 2012_2, concatenated with a list of commonly observed background contaminants. For the search, oxidation of methionine, protein N-terminal acetylation, QQTGG modification of lysine and pyroglutamate-QTGG were specified as variable modifications and carbamidomethyl of cysteine was set as fixed modification. The FDR rate was set at 1% according to the decoy database search strategy [22].

### Depletion of proteins by RNAi

Cells were subjected to a double knock-down protocol using Lipofectamine RNAiMAX (Life Technologies) to deliver 10 nM of RhoGDIα (#1: s698 - CCAGCAUACGUACAGGAAA, #2: s699 - GUCUAACCAUGAUGCCUUA, #3: s700 - CCAUGAUGCCUUAACAUGU, Ambion Silencer Select, Life Technologies), RhoGDIβ (#1: s455 - GGAAGGUUCUGAAUAUAGA, #2: s456 - CCAUGGACCUUACUGGAGA, #3: s224014 - GUGGAUAAAGCAACAUUUA, Ambion Silencer Select, Life Technologies), Ubc9 (stealth predesigned UBE2IHSS111130 - UCGAACCACCAUUAUUUCACCCGAA, Life Technologies) or 100 nM PIAS1-4 (PIAS1 – GGAUCAUUCUAGAGCUUUA, PIAS2 – CUUGAAUAUUACAUCUUUA, PIAS3 – CCCUGAUGUCACCAUGAAA, PIAS4 – GGAGUAAGAGUGGACUGAA) [23] siRNA oligos. As a control, 100 nM of Luciferase (CGUACGCGGAAUACUUCGA, Sigma) siRNA oligo was used. Cells were transfected 48 and 24 hours before harvest.

### *In vivo* SUMOylation immunopurification assay

Stable HeLa FRT TRex cell lines were induced with doxycycline and pre-synchronized with a thymidine block before a final arrest overnight in mitosis with taxol. Mitotic cells were harvested by shake-off and lysed in lysis buffer (50 mM Tris pH 7.5, 150 mM NaCl, 1 mM EDTA, 1% NP-40) supplemented with 2 ng/ml Microcystin-LR (Enzo Life Sciences), 1 mM DTT, 20 mM NEM, and EDTA-free protease inhibitor cocktail (Roche). The cleared lysate was added to GFP-trap (ChromoTek) beads and the mixture was incubated at 4°C for 1 hour. The beads were washed twice in lysis buffer and resuspended in 2×LDS sample buffer and boiled for 10 min at 95°C for elution of proteins captured by the GFP-trap. Samples were analyzed by western blotting the same day as the immunopurification to avoid de-SUMOylation of targets.

### Time lapse analysis

Live cell analysis was performed on a Deltavision Elite system (GE Healthcare) using a 40× oil objective. Cells were seeded in 8 well Ibidi dishes (Ibidi) in advance and prior to filming the media was changed to Leibovitz's L-15 (Life technologies). Appropriate channels were recorded for 18 hours and data analyzed using Softworx (GE Healthcare).

## Results

### A strategy to efficiently purify SUMO2/3 modified proteins from mitotic cells

Given the importance of the SUMO pathway for proper execution of mitosis, we wanted to determine the proteins being modified in an unbiased manner. We focused our efforts on SUMO2/3 as this modification is known to be more dynamic than SUMO1 during mitosis [13].

**Figure 1:**
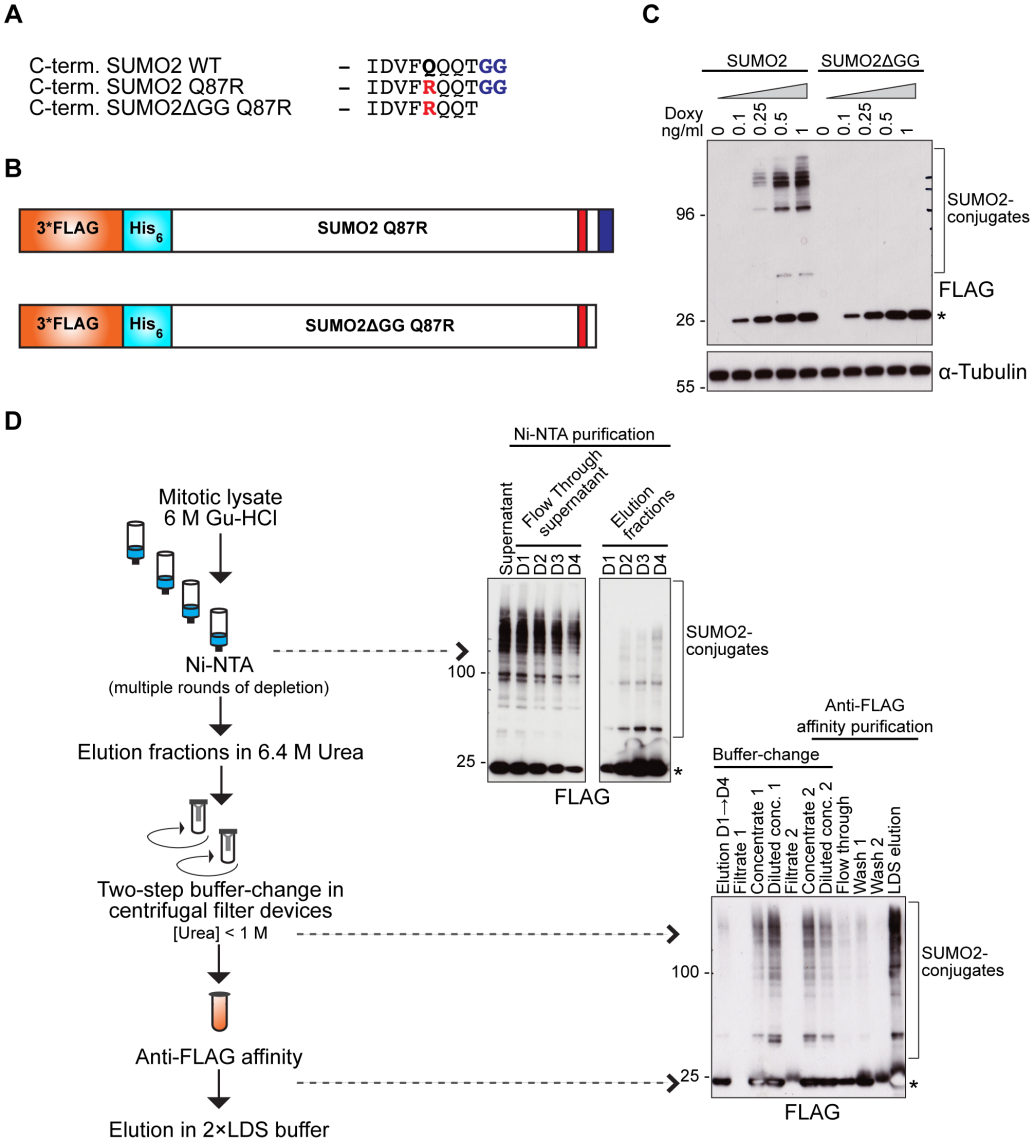
Enrichment of SUMO2/3-conjugates through tandem-affinity purification. A) Sequence of the C-terminus of mature SUMO2 and the two mutants, SUMO2 Q87R and SUMO2ΔGG Q87R. The Q to R mutation is highlighted in red and the di-glycine conjugation motif in blue. B) Schematic representation of the generated SUMO2 Q87R and SUMO2ΔGG Q87R fusion constructs with the N-terminal 3*FLAG-His double affinity tag. C) Expression of SUMO2 fusion proteins were gradually induced with increased concentrations of doxycycline (ng/ml) added for 24 hours. SUMO2 fusion protein was conjugated to target proteins, whereas SUMO2ΔGG was not. (*) indicates un-conjugated SUMO2 fusion proteins. D) Schematic outline of the double-affinity purification procedure. Examples of the steps in Ni-NTA purification, buffer-change and anti-FLAG affinity purification are also shown on western blots with anti-FLAG primary antibody that detects SUMO2 fusion proteins. D1 = depletion 1. (*) indicates free 3*FLAG-His-SUMO2 fusion protein.

**Figure 2:**
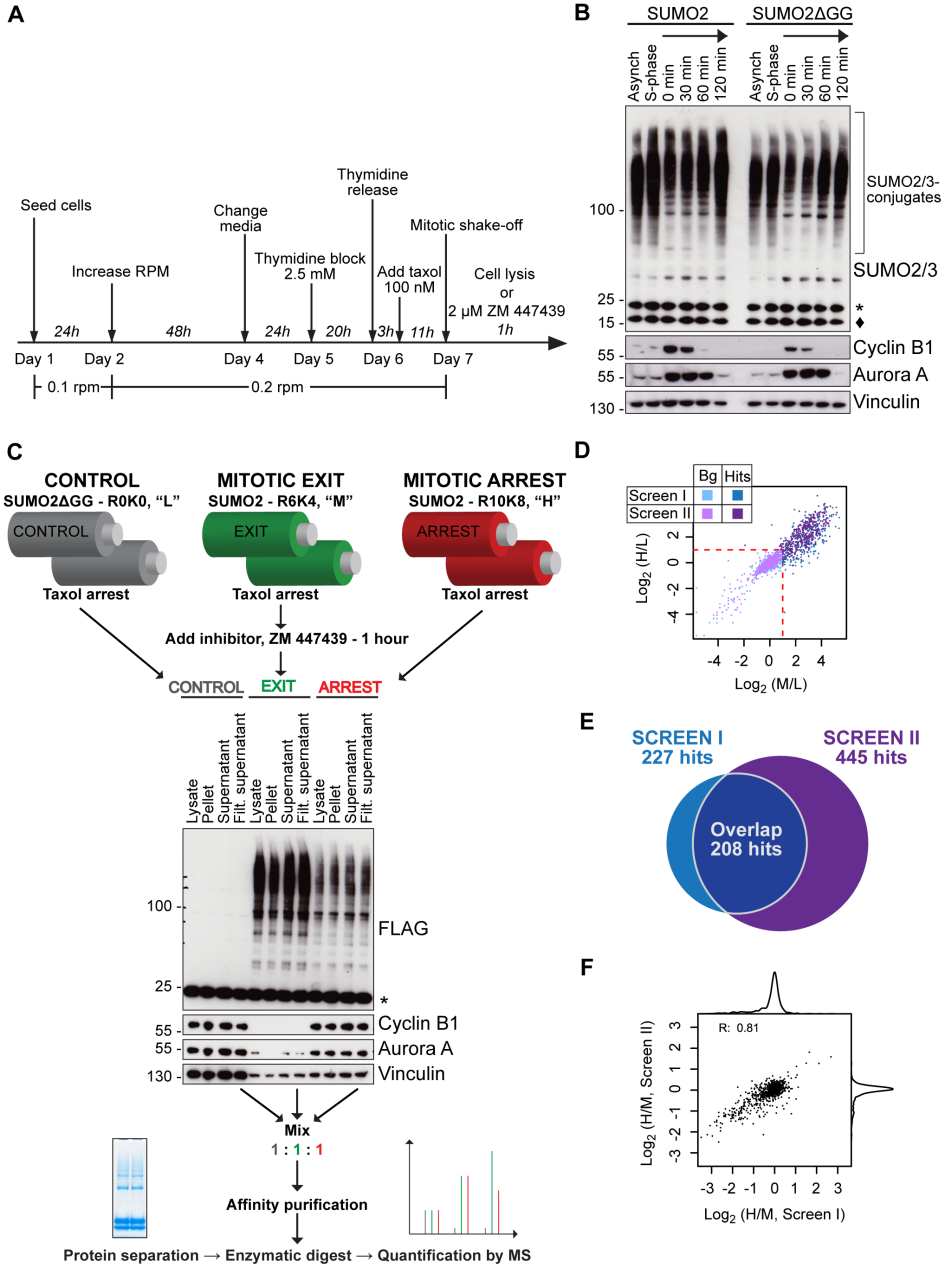
Identification of mitotic SUMOylation targets using quantitative proteomics. A) Schematic outline of the protocol for growth and synchronization of cells in roller bottles. B) HeLa FRT TRex SUMO2 and HeLa FRT TRex SUMO2ΔGG cells were arrested in S phase by thymidine or synchronized in mitosis with thymidine and taxol, followed by provoking mitotic exit by the addition of ZM447439 for the indicated times. Cell lysates were analyzed by western blotting using antibodies against SUMO2/3, Cyclin B1 and Aurora A as markers for mitotic progression, and Vinculin. (^★^) indicates un-conjugated SUMO2 fusion proteins, (^⧫^) indicates endogenous SUMO2/3. C) Schematic outline of the quantitative proteomics strategy. Cells are grown in large-scale and synchronized in roller bottles as shown in A. SUMO2ΔGG cells were isotopically labelled with light “L”/R0K0 amino acids and arrested in prometaphase (grey), cells expressing SUMO2 were labelled with medium “M”/R6K4 amino acids and represented the mitotic exit stage one hour after ZM447439 addition (green), and cells expressing SUMO2 were labelled with heavy “H”/R10K8 amino acids and arrested in prometaphase (red). Equal amounts of cell lysate from the labelled populations were mixed and SUMO2 conjugated proteins were purified as described in Figure 1. The SUMO2 conjugate enriched sample was separated by SDS-PAGE, digested with trypsin and analyzed by mass spectrometry (MS). Lysates from the different experimental conditions were analyzed by western blotting antibodies against FLAG, Cyclin B1, Aurora A and Vinculin to confirm conjugation state and mitotic stage. (^★^) indicates un-conjugated SUMO2 fusion proteins. D) Scatter plot of the entire data set from screen I and screen II. The plot is showing the value of log_2_(M/L) and log_2_(H/L) SILAC ratios that are used to identify SUMOylation targets. The red dashed line at log_2_(M/L) = 1 and log_2_(H/L) = 1 represents the cut-off ratio of ≥2 for the respective SILAC pairs. Each point represents a single identified protein, proteins identified in screen I are illustrated in blue and proteins identified in screen II are in purple. Identified proteins that are classified as SUMOylation hits are above the dashed cut-off line and are darker colored, whereas identified proteins classified as background is below the cut-off and lighter. E) Diagram showing the number of identified targets (hits) of SUMO2/3 modification in screen I (blue), screen II (purple) and the overlap (dark blue) of hits that are identified in both. F) Scatter plot with the correlation between the screen I and screen II log_2_(H/M) ratios of identified SUMO2/3 target proteins. Each point represents a SUMO2/3 target. The pearson correlation, R, is shown.

To purify SUMO2/3 modified proteins, we generated stable inducible HeLa cell lines expressing FLAG-His tagged SUMO2 in addition to endogenous SUMO2 (Figure 1A and 1B). All our SUMO2 constructs contained the Q87R mutation. This improves the identification of the exact SUMO2 conjugation site on target proteins by mass spectrometry (MS) by reducing the complexity of the added SUMO chain [24], [25]. To aid simplicity constructs are simply referred to as SUMO2 throughout. The related cell line expressing FLAG-His-SUMO2ΔGG, with a deletion of the C-terminal di-glycine conjugation motif, was generated to distinguish between specific and unspecific binding in the subsequent affinity purification steps. The exogenous SUMO2 and SUMO2ΔGG proteins were expressed at close to endogenous levels after doxycycline induction and importantly, their expression did not appear to grossly change the SUMO2/3 modification pattern in cells (see Figure 2B for endogenous versus exogenous levels). Tagged SUMO2 was conjugated to target proteins whereas SUMO2ΔGG was not, as expected (Figure 1C).

Purification of SUMO2 conjugated target proteins was performed using the N-terminal double-affinity tag (Figure 1D). The purification protocol was optimized to reduce the amount of background binding to the affinity resins used. We found that a tandem affinity purification protocol, consisting of Ni^2+^-affinity purification under denaturing conditions, followed by a buffer-change step with the purpose of lowering the concentration of urea, and then a second purification step using anti-FLAG affinity gave the best results (Figure 1D). The binding of tagged SUMO2 to the Ni^2+^ affinity resin was inefficient from mammalian cell extracts. Therefore, to increase the yield of purified SUMO2/3-conjugates, the flow through lysate was passed over the Ni^2+^ affinity resin multiple times and bound proteins pooled.

Although pilot screens confirmed the efficiency of our purification strategy, large amounts of synchronized cells were needed to robustly identify SUMO2/3 modified proteins by MS. By growing cells in roller bottles, this was possible and easy to combine with standard synchronization procedures (Figure 2A). To synchronize cells in mitosis the microtubule stabilizing compound taxol was added for 11 hours and cells collected by mitotic shake-off. FACS analysis of the tagged SUMO2 cell line revealed that this synchronization protocol resulted in a G2/M arrest (Figure S1A and Materials and Methods S1) and visual inspection of cells by microscopy revealed that they were arrested in prometaphase. The addition of taxol to the medium arrests cells in prometaphase by activating the spindle assembly checkpoint [26]. A very efficient and synchronous mitotic exit was achieved from taxol-arrested cells by inhibition of Aurora B with ZM447439 [27], as revealed by the rapid disappearance of Cyclin B1 and Aurora A already one hour after inhibitor addition (Figure 2B). Inducing synchronous mitotic exit in this way has the advantage of providing a very homogenous cell population. The level of SUMO2/3 modified proteins is increased as cells exited mitosis, in agreement with previous observations (Figure 2B) [13].

We have thus established an efficient tandem affinity purification protocol for SUMO2/3 modified proteins and a method for obtaining large quantities of synchronized cells in mitosis and upon exit.

### A quantitative mass spectrometry screen for SUMO2/3 modified proteins during mitosis and upon exit

We then combined our SUMO2/3 purification setup with stable isotope labeling in cell culture (SILAC) quantitative MS [28], [29]. Three distinct sets of isotopically labeled arginine and lysine amino acids were used to label cell cultures, allowing discrimination of peptides from the different cultures in the mass spectrometer (Figure 2C). The SUMO2ΔGG cell line was grown in light (“L”, R0K0) medium and arrested with taxol, and served as control. For the mitotic arrest state, the SUMO2 cell line was grown in heavy (“H”, R10K8) medium and arrested in prometaphase with taxol. Finally for the mitotic exit state, SUMO2 cells were grown in medium (“M”, R6K4) medium, taxol arrested and treated with ZM447439 for one hour. Incubation with ZM447439 for one hour was selected based on the timing of Cyclin B1 and Aurora A degradation (Figure 2B and 2C). The SILAC approach allows us to identify prometaphase targets (H/L ratio), mitotic exit targets (M/L ratio) and the dynamics of the SUMO2/3 modification (H/M ratio).

Equal amounts of cell extract from each cell line were mixed and SUMO2/3 conjugates were then purified from the pooled lysate (approximately 120 mg of total extract). Following separation by SDS-PAGE, the gel lane was cut into 7 slices and in-gel digested before MS analysis and quantification. Two independent replica purifications were performed (screen I and screen II) and only proteins with H/L or M/L ratios higher than 2 and with a minimum of 2 ratio counts were considered as SUMO2/3 targets. This data filtering resulted in the total identification of 227 and 445 SUMO2/3 modified proteins in screens I and II, respectively (Figure 2E and File S1). We were unable to detect any specific conjugation sites in any of the targets, despite the fact that we have used the SUMO2 Q87R mutation. Although we do not know the exact reason for the difficulty in identifying sites of SUMO2/3 conjugation, we suspect that it is due to the generally low amounts of SUMO2/3 conjugates in the samples, despite our strenuous efforts to preserve them. In general it is difficult to identify sites of conjugation as often only a small fraction of a given protein is modified. Of the 227 proteins identified in screen I, 208 were also identified in screen II showing good reproducibility in identification. Individual SILAC ratios identified in the two replica screens also show a good correlation, with a correlation coefficient in the range from 0.81 to 0.9 (Figure 2F and Figure S1B–C), which confirms the robustness of the obtained data. Based on the overlap between screens and good correlation coefficients the majority of the SUMO2/3 targets identified in screen II are likely true. We identified all components of the SUMO conjugation pathway and all of the PIAS SUMO ligases, as well as previously reported mitotic targets such as Topoisomerase IIα and NuMA, validating our approach.

**Figure 3:**
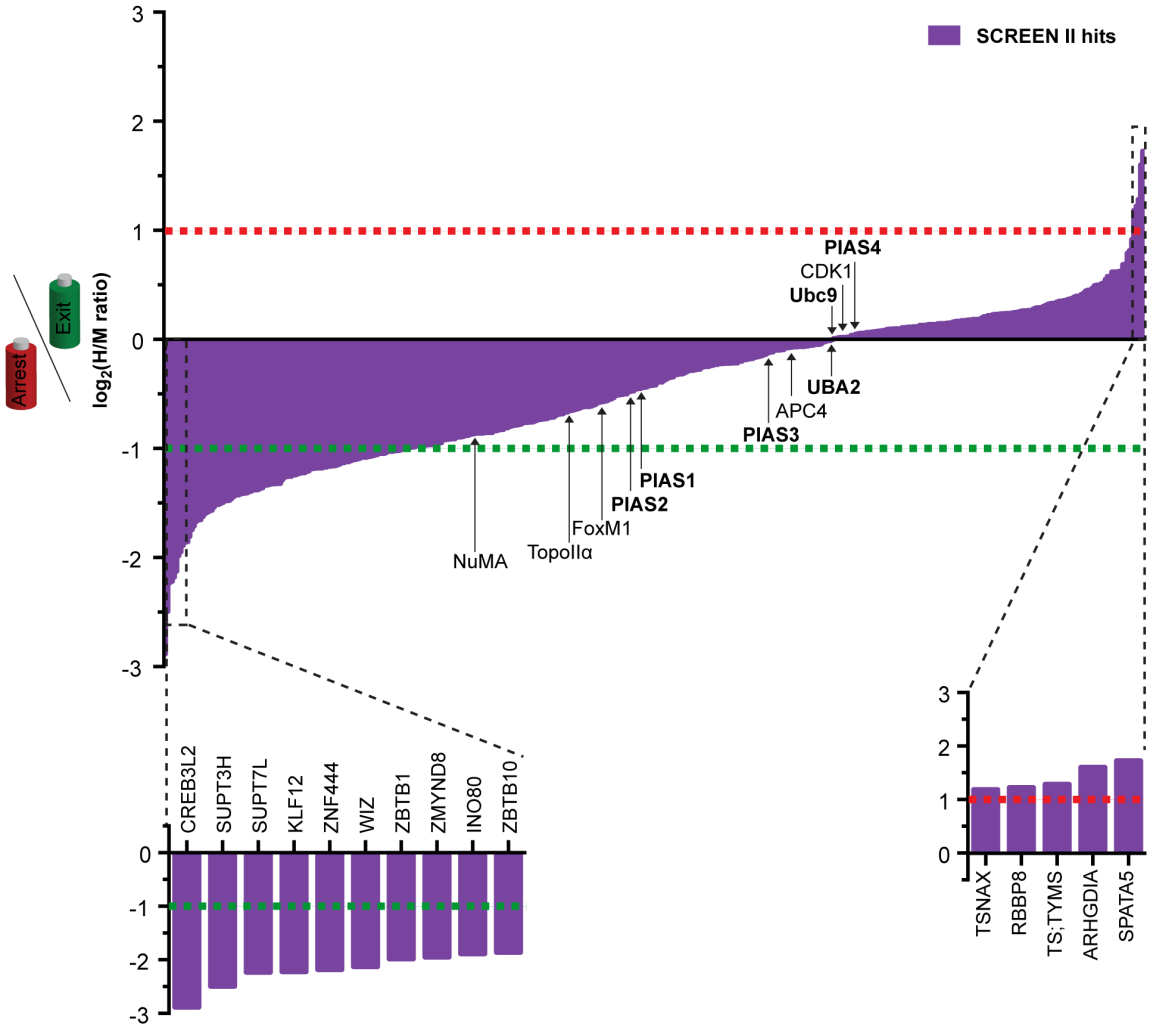
Dynamics of SUMO2/3 modifications in mitosis. Graph showing the individual modification dynamic, log_2_(H/M), of the 445 SUMO2/3 targets identified in screen II (purple). The dashed lines represent the |log_2_(H/M)|≥1 cut-off for modification dynamics. Below the green line at log_2_(H/M) = −1, targets are SUMOylated more in mitotic exit, and above the red line at log_2_(H/M) = 1, targets are SUMOylated in prometaphase arrest and deSUMOylated upon mitotic exit. Targets between the two lines are modified by SUMO at a constant level through mitosis or are showing dynamic to a lesser extent. The 10 most dynamic targets that are modified upon mitotic exit and the 5 identified targets that preferably are SUMOylated in prometaphase are highlighted below the graph with their respective gene names. Names of identified SUMO pathway components (in bold) and known targets of SUMOylation in mitosis are shown, and arrows indicate their respective ratios.

To gain insight into individual target - and overall – SUMO2/3 modification dynamics in mitosis, we next used the H/M SILAC ratios of the classified SUMO2/3 targets from screen II that were identified by MS. Based on the log_2_(H/M) value, the targets were divided into three groups; increased SUMO2/3 modification upon exiting a prometaphase arrest (log_2_(H/M)≤−1, n = 110), increased SUMO2/3 modification during prometaphase (log_2_(H/M)≥1, n = 5) or constantly modified by SUMO2/3 in mitosis (−1<log_2_(H/M)<1, n = 330) (Figure 3A). This reveals that there is a large increase in proteins getting SUMO2/3 modified upon exit from mitosis in agreement with the total increase in SUMO2/3 modified proteins we see by western blot (Figure 2B and 2C). Surprisingly, few proteins that are specifically modified during prometaphase were detected.

### RhoGDIα is specifically modified by SUMO2/3 in prometaphase

**Figure 4:**
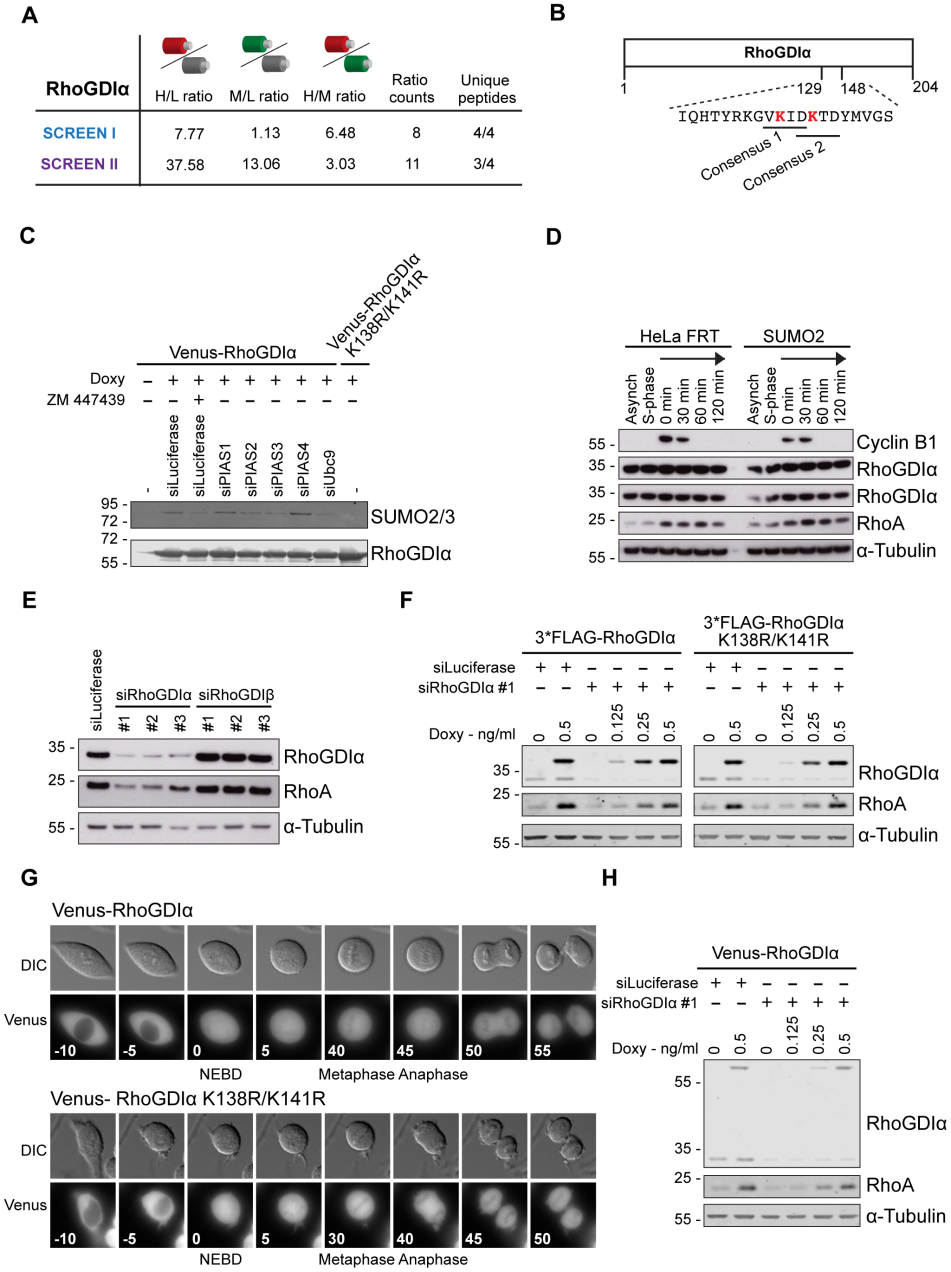
RhoGDIα is specifically modified by SUMO in prometaphase. A) Identification of RhoGDIα as a SUMO2/3 target. SILAC ratios and MS data from screen I and II. B) Schematic representation of RhoGDIα with the amino acid sequence around lysine residues K138 and K141. The consensus modification motifs are indicated and the lysines are highlighted in red. C) Purification of Venus-RhoGDIα or Venus-RhoGDIα K138R/K141R from taxol arrested cells using GFP-trap beads. SUMO conjugation pathway components, Ubc9 and PIAS1-4, were depleted by RNAi, expression of Venus-RhoGDIα fusion proteins in the generated stable HeLa FRT Trex cell lines was induced with doxycycline and ZM447439 was added for one hour as indicated. The SUMOylation states of purified RhoGDIα and RhoGDIα K138R/K141R from the different experimental conditions were analyzed by western blotting with antibodies specific for SUMO2/3 and RhoGDIα. D) Parental HeLa FRT and HeLa FRT TRex SUMO2 cells were arrested in S phase by thymidine or synchronized in mitosis with thymidine and taxol, followed by mitotic checkpoint override and progression by the addition of ZM447439 for the indicated times. Cell lysates were analyzed by western blotting using antibodies against Cyclin B1, RhoGDIα, RhoA and α-tubulin, and shows that RhoGDIα itself is stable in mitosis. E) Using 3 different siRNA oligos for each target, normal HeLa cells were depleted for RhoGDIα or RhoGDIβ using RNAi. Lysates were analyzed for depletion efficiency and RhoA stabilization by western blot with RhoGDIα and RhoA specific antibodies. F) Stable HeLa FRT TRex FLAG-RhoGDIα or FLAG-RhoGDIα K138R/K141R cells were depleted for endogenous RhoGDIα and arrested in mitosis with taxol. Rescue with exogenous RhoGDIα fusion proteins were titrated in with increasing concentrations of doxycycline (ng/ml) as indicated. Lysates were analyzed for depletion efficiency, expression level of exogenous RhoGDIα and RhoA stabilization by western blot with RhoGDIα and RhoA specific antibodies. G) Representative still images from time-lapse movies of stable HeLa cell lines expressing Venus-RhoGDIα and Venus-RhoGDIα K138R/K141R as they progress through an unperturbed mitosis. The DIC and Venus channels are shown and the time of nuclear envelope breakdown (NEBD), metaphase and anaphase is indicated. H) As F) but using Venus-RhoGDIα.

The Rho GDP dissociation inhibitor, RhoGDIα, was robustly identified as a target of SUMO2/3 modification in both screens and was one of the five targets that exhibited a modification in taxol-arrested cells which was subsequently reduced upon mitotic exit (Figure 4A). Given the specificity of RhoGDIα SUMO2/3 modification during a taxol arrest we focused on this target, as we speculated that the SUMO2/3 modification could influence changes in RhoA activity required for proper mitotic progression. To validate the MS identified SUMO2/3 modification of RhoGDIα, and indirectly our screen, we generated a stable HeLa cell line expressing Venus-tagged RhoGDIα. Venus-RhoGDIα was purified from taxol-arrested cells or taxol-arrested cells treated with ZM447439 for one hour. In the taxol-arrested cells, we indeed observed a single SUMO2/3 specific band that correlated in size with a mono-SUMOylated form of Venus-RhoGDIα (Figure 4C). On long exposures of RhoGDIα western blots of whole cell extracts from synchronized cells, we also observed a slower migrating band corresponding in size to SUMO2/3 modified endogenous RhoGDIα. This band appeared specifically in taxol-arrested cells and disappeared upon mitotic exit (Figure S1D). Although we have been unable to confirm the exact nature of this slower migrating band, it is likely SUMO2/3 modified RhoGDIα as it follows the same dynamics as we observe in the MS screen. The modification was absent in the purification from ZM447439 treated cells, in agreement with our screen and in addition depended on the presence of Ubc9 (Figure 4C). As the protein level of RhoGDIα was constant under our conditions (Figure 4D), the observed modification dynamics are likely due to a change in the balance of SUMO ligase/deSUMOylase activities. We sought to identify the lysine residues to which SUMO2/3 was conjugated. RhoGDIα can be modified with SUMO1 on K138 [30] and SUMOylation site prediction using SUMOsp [31] identified K141 as another potential conjugation site (Figure 4B). Both residues are on the surface of RhoGDIα and when we mutated both to arginine SUMO2/3 modification was abolished in taxol-arrested cells (Figure 4C). To determine the E3 ligases involved in SUMO2/3 modification of RhoGDIα we used previously reported functional RNAi oligos targeting PIAS1-4 [23] and depleted these ligases from the stable Venus-RhoGDIα cell line. This revealed that both PIAS2 and PIAS3 were required for efficient SUMO2/3 modification of RhoGDIα during a taxol arrest (Figure 4C).

When the stability of RhoGDIα was analyzed, we noticed that the level of RhoA was increased in mitosis (Figure 4D). It is clear that RhoA is stabilized and protected from degradation by RhoGDI binding, and that the stabilization is specifically mediated through RhoGDIα and not RhoGDIβ in HeLa cells (Figure 4E) [32]. The increase in RhoA levels in prometaphase correlates with the timing of SUMO2/3 modification of RhoGDIα. To investigate if SUMO2/3 modification of RhoGDIα contributes to the increased RhoA stability during mitosis, we depleted endogenous RhoGDIα by RNAi in inducible stable cell lines expressing FLAG-RhoGDIα or FLAG-RhoGDIα K138R/K141R that were siRNA resistant. RhoA was equally well stabilized by both forms of RhoGDIα, arguing that SUMO2/3 modification of RhoGDIα is not critical for RhoA stability during mitosis (Figure 4F). We also analyzed the localization of Venus-RhoGDIα and Venus-RhoGDIα K138R/K141R by time-lapse microscopy as cells progressed through mitosis. Neither the wild-type nor mutant Venus-RhoGDIα localized to any distinct mitotic structures. Instead, both were evenly distributed throughout the mitotic cell. It is possible that we could not detect any difference in localization between the two forms of RhoGDIα because only a small pool is SUMO2/3 modified (Figure 4G). The Venus tagged proteins appeared functional in that they could stabilize RhoA (Figure 4H).

Our analysis of RhoGDIα SUMO2/3 modification confirmed our MS data and revealed that both PIAS2 and PIAS3 are required for SUMO2/3 conjugation to K138 and/or K141. The exact role of SUMO2/3 modification of RhoGDIα during mitosis is unclear but our data does not support a role in mitotic RhoA stabilization.

## Discussion

We have here provided a comprehensive characterization of proteins modified by SUMO2/3 during a prometaphase arrest and how this changes when cells exit mitosis. This provides an in-depth analysis of SUMO2/3 targets during mitosis, providing a framework for dissecting the role of this modification in mitosis.

### Regulation of mitotic progression by SUMO2/3 modifications

Previously, different proteomic approaches have been used to identify targets of SUMOylation, but often the identification has been performed under cellular conditions where SUMOylation is high such as heat shock [15], [33], [34] or when proteasomal degradation has been inhibited [35], [36]. The methods we have developed here allow robust identification under cellular conditions where SUMO2/3 modified proteins are of low abundance. Critical for efficient detection is a simple and efficient purification strategy, and the sequential use of a His-tag followed by a FLAG affinity step proved to be very efficient. An alternative approach is the use of protein arrays and by applying mammalian mitotic cell extracts to such an array, another study identified approximately 500 SUMO2/3 targets [37]. A comparison with our data set revealed limited overlap potentially reflecting the very different experimental approaches. A recent proteomic screen using FLAG affinity purification and synchronization with CDK1 inhibition [38] showed some overlap with our data (see File S1). In the screen by Schimmel et al. a total of 73 mitotic targets were identified, of which we identified 25. In contrast to that study, which employed overexpression of tagged SUMO2, we express tagged SUMO2 at close to endogenous levels. Despite this, our purification and identification strategy identified markedly more mitotic targets, more than 400 in all, suggesting it is efficient in enriching and identifying SUMO substrates.

Although detecting SUMO modified proteins is of interest, the dynamic changes we map here provide an additional layer of information that can help to identify modifications that play regulatory roles. Our screen confirmed that there is an increase in proteins becoming modified by SUMO2/3 upon mitotic exit, while fewer proteins are specifically modified during mitosis, which is in agreement with previous observations [13]. Many of the proteins getting strongly modified upon mitotic exit are linked to transcription, in line with the reported regulatory role of the SUMO pathway in this biological process [39], [40]. Due to the compact nature of chromatin during mitosis, transcription is limited, but upon exit transcription resumes and likely the observed SUMO2/3 modification on some of these proteins regulates transcription.

In addition, numerous proteins involved in mitotic regulation such as NuMA, RanGAP1, Topoisomerase IIα, CDK1 and APC4 were identified, although the change in SUMO2/3 modification was less on these proteins. KNL2 (Mis18BP1) was an interesting dynamic SUMO2/3 target we identified. KNL2 is required for CENP-A loading upon mitotic exit [41]–[43] and this correlates with its modification by SUMO2/3. In addition, Repo-Man, a regulator of Aurora B activity [44], [45], displayed increased SUMO2/3 modification upon treatment with ZM447439. The role of SUMO2/3 modification of these proteins will be interesting to explore.

### RhoGDIα SUMOylation in mitosis

RhoGDIα was robustly identified in our screen as a protein modified by SUMO2/3 during a taxol induced mitotic arrest but not upon mitotic exit. We used this target both as a means to validate our screen data by a non-MS approach and also to demonstrate that RhoGDIα is likely modified by a single SUMO2/3 molecule on either K138 or K141. RNAi depletion of the different PIAS SUMO E3 ligases showed that both PIAS2 and PIAS3 were required for efficient SUMO2/3 modification. Whether the requirement for both PIAS2 and PIAS3 reflects the possibility that they exist in a complex is presently unclear.

RhoGDIα has previously been shown to be modified by SUMO1 on K138. This has been suggested to increase its affinity for Rho GTPases, like RhoA, in order to negatively regulate their activity and decrease cell migration [30]. Whether the modification of RhoGDIα by SUMO2/3 we identify here in prometaphase negatively regulates RhoA is unclear, but we have been unable to detect a role of this modification in stabilizing RhoA, which is another known function of RhoGDIs [32]. RhoA activity [46] and levels (our data) are increased during mitosis, which potentially could be regulated by RhoGDIα. However, we have not detected any mitotic defects by time-lapse microscopy in cells efficiently depleted of RhoGDIα, arguing against this hypothesis. Potentially the SUMO2/3 modification of RhoGDIα is a response to the detachment from the surface that occurs during mitosis and therefore might not play a direct role in mitotic processes.

Combined, we have here provided an in-depth analysis of SUMO2/3 modified proteins during mitosis that will be useful for addressing precisely how the SUMO pathway coordinates mitotic progression.

## Supporting Information

Figure S1 **Cell synchronization and correlation between screens.** A) FACS profiles of the stable HeLa cell line expressing tagged SUMO2 Q87R used for purification of conjugates. The FACS profile of asynchronous cells and cells synchronized with taxol are shown and the percentage of cells in G1 and G2/M is indicated. B) Scatter plot with the correlation between the screen I and screen II log_2_(H/L) ratios of identified SUMO2/3 target proteins. C) Scatter plot with the correlation between the screen I and screen II log_2_(M/L) ratios of identified SUMO2/3 target proteins. Each point represents a SUMO2/3 target. The pearson correlation, R, is shown. D) HeLa FRT TRex SUMO2 cells were arrested in S phase by thymidine or synchronized in mitosis with thymidine and taxol, followed by mitotic checkpoint override and progression by the addition of ZM447439 for the indicated times. Cell lysates were analyzed by western blotting using antibodies against Cyclin B1, RhoGDIα and α-tubulin. A short and long exposure of the RhoGDIα blot is shown and the putative RhoGDIα-SUMO2/3 species is indicated.
(TIF)

File S1 **List of all SUMO2 targets identified in this study. Related to** **Figure 2** **and** **3** **.** The excel-file contains all the proteins that have been classified as SUMO2/3 targets in screen I and screen II.
(XLSX)

Materials and Methods S1 **Method used for FACS experiments in Figure S1.**
(DOCX)
