# Supplementary figures and images for: Comprehensive Identification of SUMO2/3 Targets and Their Dynamics during Mitosis
Source: PLoS One. 2014 Jun 27;9(6):e100692. doi: 10.1371/journal.pone.0100692 (PMC4074068; doi:10.1371/journal.pone.0100692)

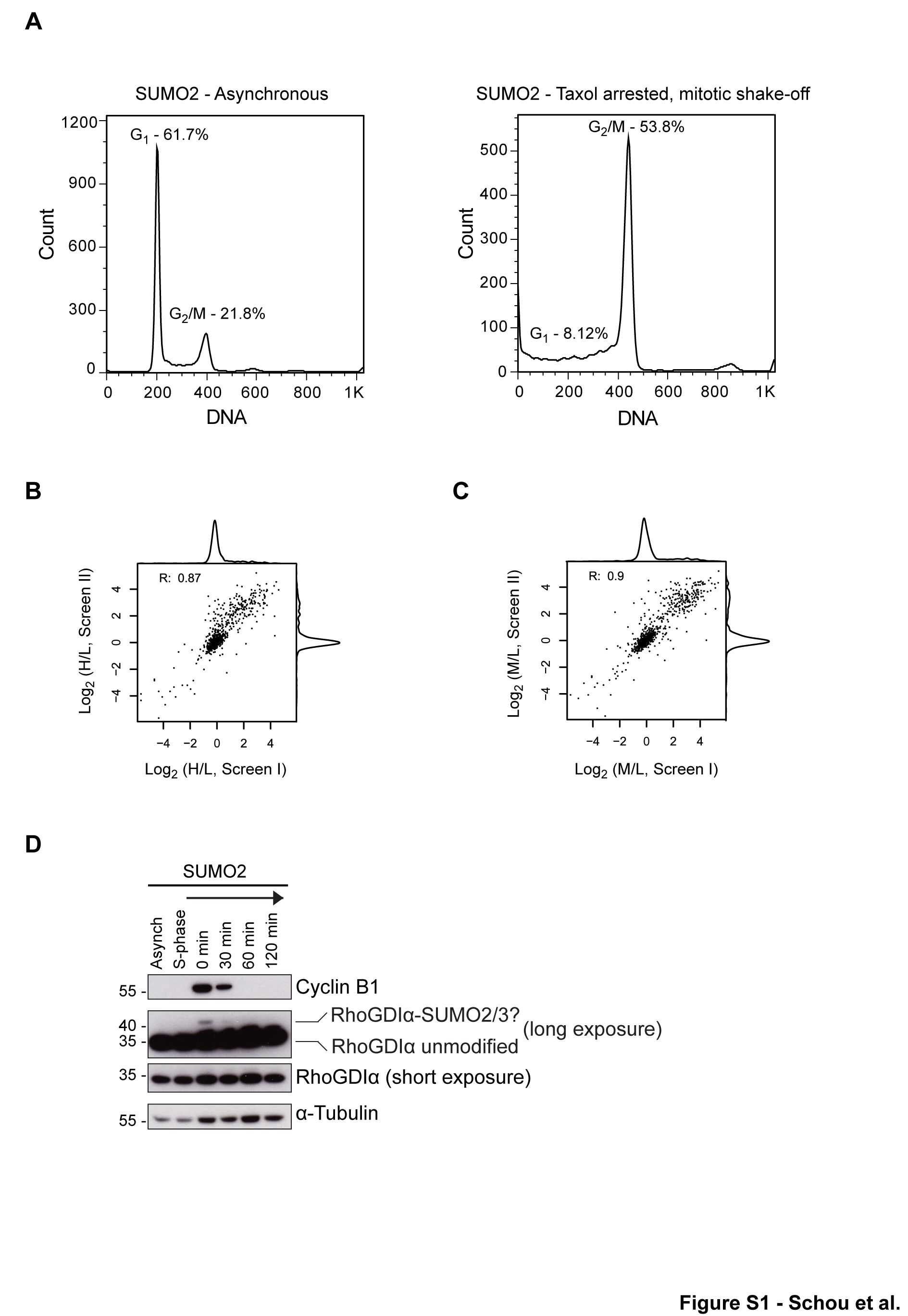

Supplement: Figure S1 — Cell synchronization and correlation between screens. A) FACS profiles of the stable HeLa cell line expressing tagged SUMO2 Q87R used for purification of conjugates. The FACS profile of asynchronous cells and cells synchronized with taxol are shown and the percentage of cells in G1 and G2/M is indicated. B) Scatter plot with the correlation between the screen I and screen II log2(H/L) ratios of identified SUMO2/3 target proteins. C) Scatter plot with the correlation between the screen I and screen II log2(M/L) ratios of identified SUMO2/3 target proteins. Each point represents a SUMO2/3 target. The pearson correlation, R, is shown. D) HeLa FRT TRex SUMO2 cells were arrested in S phase by thymidine or synchronized in mitosis with thymidine and taxol, followed by mitotic checkpoint override and progression by the addition of ZM447439 for the indicated times. Cell lysates were analyzed by western blotting using antibodies against Cyclin B1, RhoGDIα and α-tubulin. A short and long exposure of the RhoGDIα blot is shown and the putative RhoGDIα-SUMO2/3 species is indicated. (TIF) [file pone.0100692.s001.tif]
