# Supplementary material for: Comprehensive Identification of SUMO2/3 Targets and Their Dynamics during Mitosis
Source: PLoS One. 2014 Jun 27;9(6):e100692. doi: 10.1371/journal.pone.0100692 (PMC4074068; doi:10.1371/journal.pone.0100692)
Supplement: Materials and Methods S1 — Method used for FACS experiments in Figure S1. (DOCX) [file pone.0100692.s003.docx]

**MATERIALS AND METHODS S1**

**FACS cell-cycle profiles**

Asynchronous SUMO2 cells were harvested by trypsinization and SUMO2 cells arrested with thymidine and taxol as previously described were harvested by mitotic shake-off. Cells were washed twice in cold PBS, resuspended in 300 μl PBS and fixed by addition of 700 μl ice cold (-20°C) methanol while vortexing. After incubation in the dark at 4°C over night, cells were washed with PBS and resuspended in Propidium iodide buffer (0.1 mg/ml Propidium iodide (Sigma) in FACSFlow buffer (BD Biosciences)) and supplemented with 25 μg/ml RNase A (Sigma).

Cell-cycle profiles were determined by flow cytometric analysis of the propidium-iodide-stained cells using a FACSCalibur flow cytometer (BD Biosciences).
